# TOPO3α Influences Antigenic Variation by Monitoring Expression-Site-Associated *VSG* Switching in *Trypanosoma brucei*

**DOI:** 10.1371/journal.ppat.1000992

**Published:** 2010-07-08

**Authors:** Hee-Sook Kim, George A. M. Cross

**Affiliations:** Laboratory of Molecular Parasitology, The Rockefeller University, New York, New York, United States of America; University of Wisconsin-Madison, United States of America

## Abstract

Homologous recombination (HR) mediates one of the major mechanisms of trypanosome antigenic variation by placing a different variant surface glycoprotein (*VSG*) gene under the control of the active expression site (ES). It is believed that the majority of *VSG* switching events occur by duplicative gene conversion, but only a few DNA repair genes that are central to HR have been assigned a role in this process. Gene conversion events that are associated with crossover are rarely seen in *VSG* switching, similar to mitotic HR. In other organisms, TOPO3α (Top3 in yeasts), a type IA topoisomerase, is part of a complex that is involved in the suppression of crossovers. We therefore asked whether a related mechanism might suppress *VSG* recombination. Using a set of reliable recombination and switching assays that could score individual switching mechanisms, we discovered that TOPO3α function is conserved in *Trypanosoma brucei* and that TOPO3α plays a critical role in antigenic switching. Switching frequency increased 10–40-fold in the absence of *TOPO3α* and this hyper-switching phenotype required *RAD51*. Moreover, the preference of 70-bp repeats for *VSG* recombination was mitigated, while homology regions elsewhere in ES were highly favored, in the absence of *TOPO3α*. Our data suggest that TOPO3α may remove undesirable recombination intermediates constantly arising between active and silent ESs, thereby balancing ES integrity against *VSG* recombination.

## Author Summary

*Trypanosoma brucei*, the causative agent of African sleeping sickness, escapes the host immune response through a mechanism known as the antigenic variation. Each individual trypanosome expresses a single species of surface antigenic protein at any time yet possesses an infinite potential to express different surface antigens by transcriptional and recombinatorial switching. Periodic switching to a different antigen allows parasites to escape the antibody-mediated host immune response and causes chronic infection, eventually overwhelming the host's immune system and leading to death. DNA recombination factors are critical for the protection of chromosome integrity. One of the major antigen-switching mechanisms exploits particular recombination pathways to achieve its purpose. We have used a new switching assay to study a regulator of recombination and to demonstrate that antigenic variation is a complex mechanism balancing chromosome integrity and antigen diversity by suppressing and promoting particular recombination events. Recombination is used in evasion or virulence mechanisms by several pathogens. Exploring how *Trypanosoma brucei* manipulates the recombination machinery to gain advantage against their host will help us understand pathogenesis in various organisms and may reveal weaknesses that can be exploited to control infectivity and virulence.

## Introduction

*Trypanosoma brucei* proliferates in the bloodstream of its mammalian host and periodically escapes the antibody-mediated immune response. A single species of variant surface glycoprotein (VSG) is expressed at a given time, from among >1,000 *VSG* genes and pseudogenes [1], [2], and ∼10 million VSG molecules homogenously coat the surface of a parasite. Switching the expressed *VSG* causes antigenic variation (reviewed in [3]–[5]).

*VSG* genes are found in 15 expression sites (ESs) — polycistronic transcription units that are transcribed by RNA Polymerase I [3], [6]–[8] — of the Lister 427 strain [9]. These *VSG*s are located 40–60 kb downstream of their ES promoters and are flanked by 70-bp and telomere repeat sequences. Several expression-site-associated genes (*ESAG*s) with mostly unknown functions, and *ESAG* and *VSG* pseudogenes, are located between the promoter and the 70-bp repeat region. Only one ES is transcriptionally active at any time and the rest are silent. Many *VSG*s are found upstream of telomere repeats in minichromosomes but most are thought to reside in ‘telomere-distal’ arrays. Minichromosomal and telomere-distal *VSG*s lack promoters, but small numbers of 70-bp repeats are present upstream of these *VSG*s.

By analyzing switched variants, two major pathways of antigenic switching have been identified in *T. brucei*: *in situ* ES transcription switching and recombination-mediated switching [4], [5], [10]. *In situ* switching occurs by silencing the active ES and activating a silent ES, without DNA rearrangement [11], [12]. Recombination-mediated switching occurs mainly by gene conversion (GC) and can involve just the *VSG* or larger regions of the ES. *VSG* GC can occur by recombination between the active *VSG* and a silent ES-associated *VSG*, a minichromosomal *VSG*, or a telomere-distal *VSG* [13]–[18]. Gene conversion between larger regions can result in the duplication of an entire ES, including its *VSG* [12]. Crossover switches, where two *VSG*s are exchanged, have also been observed infrequently [19]–[22].

Deficiency of RAD51 or RAD51-3 (RAD51-related gene), or BRCA2, a mediator for RAD51 filament formation, decreased switching frequency in *T. brucei* [23]–[25]. Mre11 is essential for DNA damage response, as a sensor of double strand breaks (DSBs) that can be repaired by homologous recombination (HR) or non-homologous end joining (NHEJ) [26]–[28]. As in yeast and mammals, *T. brucei mre11* null mutants exhibited growth defects, hypersensitivity to a DNA damaging agent, and gross chromosomal rearrangements (GCR), but no detectable decrease in *VSG* switching [29], [30], indicating that, although antigenic variation shares core features with classic HR, specific roles for recombination factors in antigenic variation remain to be determined.

Mitotic crossover can be detrimental, leading to unequal exchanges. Sgs1, a RecQ family helicase in yeast, is one of the major factors that control spontaneous crossovers [31]. Sgs1 forms a complex with Top3 (type IA topoisomerase) and Rmi1 (RecQ-mediated genome instability), and plays major roles in the suppression of genome instability by influencing mitotic and meiotic recombination, replication fork stability, and telomere maintenance [32]–[38]. At least one mechanism of crossover-suppression appears to involve ‘dissolution’ of double Holliday Junction (dHJ) intermediates. Sgs1-Top3-Rmi1, also known as the RTR (RecQ-Top3-Rmi1) complex, is well conserved in humans as the BLM (Bloom mutated)-TOPO3α-BLAP75/18 (Bloom associated protein 75kDa/18kDa, or RMI1/2). Mutations in any member of the RTR complex increase recombination frequency and crossover [31], [32], [39]–[43]. Defects in the BLM pathway are associated with elevated levels of sister chromatid exchanges (SCEs), chromosomal breaks and translocations [40], [41], [44]–[46].

Crossover has rarely been observed in *VSG* switching. Suppression of crossover is intriguing because, in principle, the outcome of duplicative *VSG* conversion holds no apparent advantage over crossover events, as re-expressing a VSG, either exchanged or duplicated, will be lethal *in vivo*. Given the similarities between HR and *VSG* switching, we hypothesized that certain yeast hyper-recombination mutants could be hyper-switchers in trypanosomes. Using new recombination and *VSG* switching assays, we took advantage of a potential member of *T. brucei* Sgs1 pathway, *TbTOPO3α* (Tb11.01.1280), to get better insights on how trypanosomes employ recombination factors to control antigenic variation.

## Results

### Type 1A toposiomerase TOPO3α is conserved in *Trypanosoma brucei*

**Figure 1:**
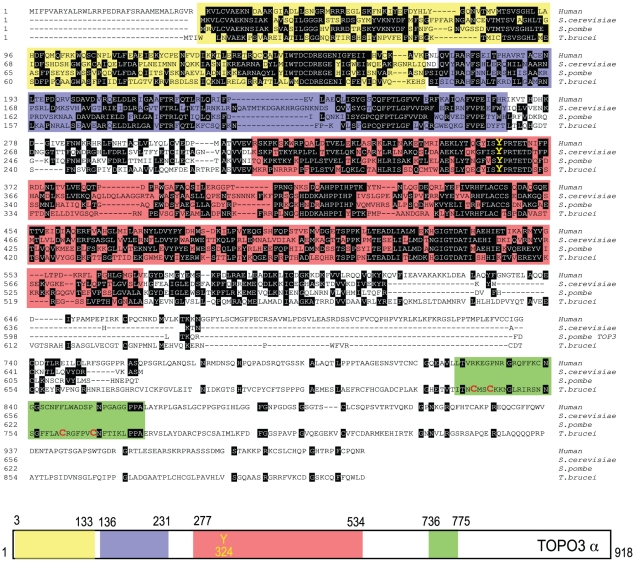
Alignment of *T. brucei* TOPO3α, human TOPO3α, *S. cerevisiae* and *S. pombe* Top3. The colored boxes indicate domains found in SMART (Simple Modular Architecture Research Tool): yellow box, TOPRIM (topoisomerase-primase) domain; purple, TOP1Bc (bacterial DNA topoisomerase I ATP-binding domain); red, TOP1Ac (bacterial DNA topoisomerase I DNA binding domain); green, Zf-C4 (zinc-finger domain). The catalytic tyrosine residue (Y) is written in yellow. Four cysteine residues are written in red in a green box.

Type IA topoisomerases cleave DNA by covalent attachment of one of the DNA strands through a 5′phosphodiester bond to a tyrosine residue in their catalytic domains [47]. In many organisms, type IA topoisomerases function in cooperation with helicases, as a combination of Top3-Sgs1 in yeasts and TOPO3α-BLM in humans. *T. brucei* expresses a 102.5-kDa TOPO3α protein with 918 amino acids. Figure 1 shows an alignment of TbTOPO3α with human TOPO3α and *S. cerevisiae* and *S. pombe* Top3. The primary sequences are well aligned at the N-terminal catalytic domain including the active site tyrosine. Both *E. coli* Top1 and human TOPO3α contain Zn-binding motif(s) in their C-terminal regions. *E. coli* Top3 and two yeast Top3 lack a Zn-binding domain (reviewed in [47]). TbTOPO3α seems to have a Zn-binding motif in the C-terminus (four cysteine residues written in red), although this region does not align well with human TOPO3α. The sequences of TOPO3α are very well conserved in *T. brucei*, *T. cruzi* and *Leishmania major* (Supporting Figure S1). *T. brucei* also has a type IA TOPO3β (http://www.genedb.org/genedb/tryp), but its function has not been studied.

### *topo3α^−/−^* exhibits a minor growth defect in *T. brucei*

To explore the role of TOPO3α, we sequentially deleted both alleles. We used deletion-cassettes containing hygromycin (*HYG*) or puromycin (*PUR*) resistance genes fused to *Herpes simplex* virus thymidine kinase (*HSVTK* or *TK*) and flanked by loxP sites, allowing the markers to be removed by transient expression of Cre-recombinase and reused [48]. Deletion of both alleles was confirmed by PCR analyses (Supporting Figure S2).

**Figure 2:**
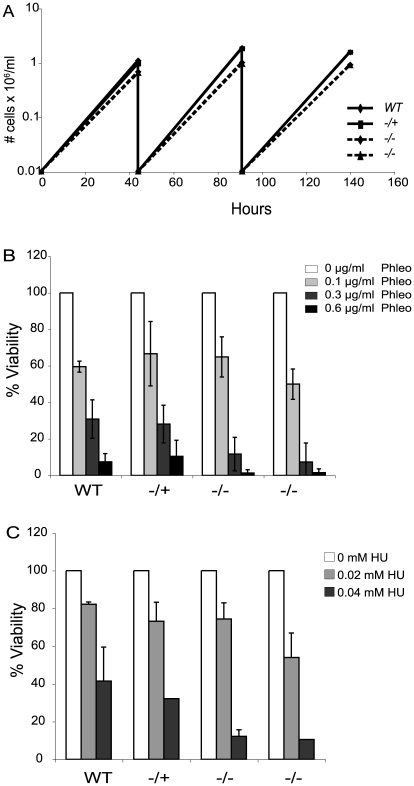
*Tbtopo3α* exhibits a minor growth defect and is sensitive to phleomycin and HU. (A) *topo3α^−/−^* shows a minor growth defect. Wild-type, *topo3α^−/+^* and *topo3α^−/−^* cells were diluted to 10,000 cells/ml and cells were counted after two days of incubation. This was repeated twice. (B) and (C) *topo3α^−/−^* is sensitive to phleomycin and HU. Cells were treated with the indicated concentrations of phleomycin (B) or HU (C) and single cells were distributed into 96-well plates. Percent viability was determined by normalizing to untreated samples. The same strains used in Figure 2A were analyzed in these experiments.

Loss of Top3 causes a severe growth defect in budding yeast and is lethal in fission yeast [43], [49]. The absence of *TOPO3α* or *TOPO3β* results in embryonic lethality or shortened life span in mice [50], [51]. In contrast, *TOPO3*α null mutants exhibited only a minor growth defect in *T. brucei* (Figure 2A).

### *Tbtopo3α* mutants are sensitive to phleomycin and hydoxyurea

Yeast Top3 is important for the maintenance of genome integrity. *top3* mutants are sensitive to DNA-damaging agents and show defects in the activation of the cell-cycle checkpoint kinase Rad53 (CHK2 in mammals), in response to genotoxic stresses [52]–[54]. We therefore asked whether *T. brucei* TOPO3α is required for the DNA damage response, by assessing sensitivity to the DSB-inducing agent phleomycin or the replication inhibitor hydroxyurea (HU). Cells were treated with phleomycin for 24 hours and single cells were distributed in 96-well plates. The color of the medium turns from red to yellow when the culture becomes saturated. Yellow wells were counted after 7–8 days and the percent viability was calculated by normalizing to the untreated samples. In the null mutants, viability was reduced by 3-fold at 0.3 µg/ml and 10-fold at 0.6 µg/ml phleomycin (Figure 2B). Viability of the HU-treated null mutants was reduced by 3-fold in 0.04 mM HU (Figure 2C). *topo3α^−/+^* was comparable to the wild type in both experiments. We conclude that TOPO3α is required for the response to DNA damage and replication block, similar to the roles of yeast Top3.

### *Tbtopo3α^−/−^* is a ‘hyper-rec’

*top3* was isolated as a hyper-recombination (‘hyper-rec’) mutant in a genetic screen designed to identify mutations that increase recombination frequency at *SUP4-o* locus in budding yeast [43]. We therefore hypothesized that *Tbtopo3α* could be a ‘hyper-rec’ mutant and this phenotype could be reflected in the frequency of recombination-mediated antigenic switching.

**Figure 3:**
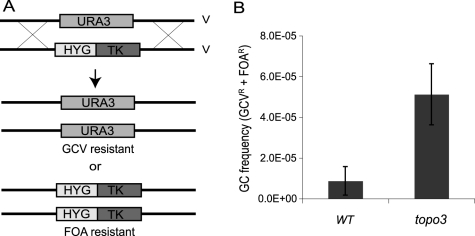
*Tbtopo3α^−/−^* is a ‘hyper-rec’. (A) Schematic diagram of recombination assay. Gene conversion frequency was determined by using two counter-selectable markers, *TK* and *URA3*. One allele of *TbURA3* was replaced with *HYG-TK*. The frequency of *HYG-TK* loss can be measured with GCV. Only the cells that had lost the *TK* gene can grow in the presence of GCV. Loss of *TbURA3* can be measured with 5-FOA, as only the *ura3^−^* cells can grow in its presence. (B) TOPO3α deficiency increases gene conversion frequency. Overall GC frequencies (*GCV^R^* and *FOA^R^*) were plotted. Error bars indicate standard deviation.

To test whether TOPO3α deficiency increases recombination frequency, we established a new recombination assay. Thus far, transfection-based recombination assays have been predominantly used, in which trypanosomes are transfected with linear DNA containing a selection marker flanked by targeting sequences, and the recombination frequency is calculated from the number of drug-resistant clones that arise. Although this method can give reliable measurements, it requires a high rate of recombination at the target site and is subject to variations in transfection efficiency. To allow a more convenient, natural and reliable measure of recombination efficiency, we established an assay (Figure 3A) in which *HYG-TK* can replace one allele of what we will call *TbURA3* (the bifunctional orotidine-5-phosphate decarboxylase/orotate phosphoribosyltransferase Tb927.5.3810) on chromosome V. The frequency of loss of either the *HYG-TK* or *TbURA3* allele represents the rate of gene conversion at this locus. The frequency of *HYG-TK* loss can be measured with gancyclovir (GCV), a nucleoside analog, as only the cells that had lost the *TK* gene can grow in the presence of GCV. The loss of *TbURA3* can be measured with 5-FOA (5-fluoroorotic acid), as only the *ura3^−^* cells can grow in the presence of 5-FOA.

To remove the *HYG-TK* and *PUR-TK* markers that were used for the deletion of *TOPO3α*, Cre-recombinase was transiently transfected into the *topo3α^−/−^* cells and *GCV^R^ HYG^S^ PUR^S^* clones were selected (Supporting Figure S2). One allele of *TbURA3* was then replaced with *HYG-TK* and the targeting was confirmed by PCR. Gene-conversion frequencies were determined by counting total *GCR^R^* and *FOA^R^* cells, in three wild-type and five *topo3α^−/−^* independent *HYG-TK* clones. As shown in Figure 3B, *Tbtopo3α* gave indeed a hyper-recombination phenotype. Total gene-conversion frequency was increased 6-fold in *topo3α^−/−^* (5.12±0.15×10^−5^) compared to the wild type (0.87±0.70×10^−5^).

### *Tbtopo3α^−/−^* is a *VSG* ‘hyper-switcher’ and this phenotype requires *RAD51*

**Figure 4:**
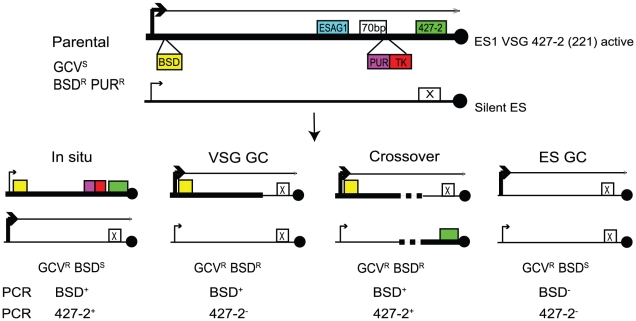
Strategies to determine the frequency and mechanisms of *VSG* switching. Parental cells have *VSG* 427-2-ES1 active. ES1 was doubly marked with *BSD* and *PUR-TK*. *PUR-TK* will either be lost (*VSG* and ES GC) or repressed (*in situ* and crossover) in switched cells. Therefore, only the switchers can grow in the presence of GCV. Switchers can later be distinguished by analyzing *VSG* 427-2 and *BSD* as specified in Table 1.

To investigate the roles for TOPO3α in *VSG* switching, we generated a *VSG* switching reporter strain in which we could easily measure switching frequency and score different switching mechanisms. As illustrated in Figure 4, the parental strain expresses *VSG* 427-2 (221) in ES1, which was doubly marked with a blasticidin-resistance gene (*BSD*) downstream of the promoter and *PUR-TK* at the 3′ end of the 70-bp repeat region, without disrupting the co-transposed region (CTR), disruption of which has been shown to induce rapid *VSG* switching [55]. The 5′ boundaries for recombination-mediated *VSG* switching have been mapped at regions upstream of CTRs that are located between the 70-bp repeats and the *VSG*. Therefore, the *PUR-TK* gene will either be lost or repressed in switched cells. This will allow switchers, but not the parental cells, to grow in the presence of GCV.

**Figure 5:**
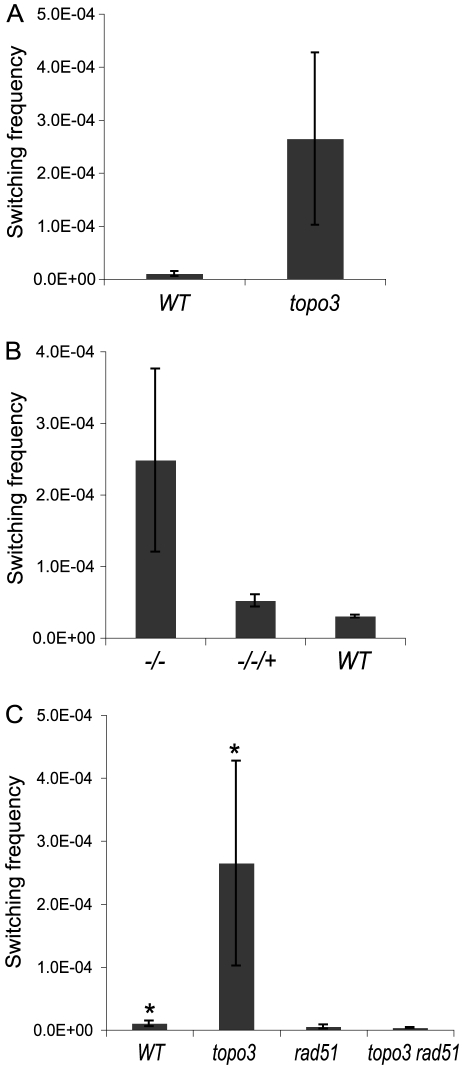
*Tbtopo3α^−/−^* is a *VSG* ‘hyper-switcher’ and this phenotype requires *RAD51*. (A) TOPO3α deficiency increased total switching frequency by 10–40-fold. Switchers were enriched as described previously [56] and selected in GCV-containing media. The switching frequency was determined by counting *GCV^R^* clones. (B) Hyper-switching phenotype is associated with TOPO3α deficiency. Switching frequency was measured in GCV-containing media without the column-enrichment. (C) Hyper-switching phenotype requires *RAD51*. Switching frequency of wild type, *topo3α^−/−^*, *rad51^−/−^* and *topo3α^−/−^rad51^−/−^* was determined as described above (Figure 5A). Error bars indicate standard deviation. (*) The same data as presented in Figure 5A.

Doubly marked wild-type and *topo3α^−/−^* cells were maintained in media containing blasticidin and puromycin, to exclude switchers from the starting population. The cells were allowed to switch in the absence of drugs for 3–4 days. Un-switched VSG 427-2-expressing cells were depleted by magnetic-activated cell sorting (MACS) [56]. The column flow-through, highly enriched with switchers, was serially diluted in medium containing 4 µg/ml GCV and distributed into 96-well plates. Switching frequency was determined as the ratio of *GCV*-resistant cells to the total number of cells prepared for the MACS column experiments. We analyzed three independent wild-type cultures and four *topo3α^−/−^* cultures. As shown in Figure 5A, TOPO3α deficiency caused a 10–40-fold increase in switching frequency (26±16×10^−5^) compared to wild type (1.01±0.45×10^−5^). This is the only known example of an increase in *VSG* switching frequency when a repair factor is deleted. To confirm that the column-mediated depletion of VSG 427-2-expressing cells was not biasing our results, other batches of cells were directly diluted in GCV-containing media and distributed into 96-well plates. Switching frequency was 10–30-fold increased in the absence of TOPO3α (data not shown).

We have determined the switching frequency in a strain without the *TK* marker but with a *PUR* marker inserted downstream of *VSG* 427-2 and obtained similar frequencies, ∼1×10^−5^, in wild type. In two different but closely related cell lines, with the same genotype except that one line has *PUR-TK* inserted at the 70-bp repeat and the other just *PUR*, again similar switching frequencies, ∼1×10^−5^, were observed [56] (personal communication with Nina Papavasiliou).

Reintroduction of wild-type *TOPO3α* complemented the hyper-switching phenotype of *topo3α^−/−^* (*−/−/+* in Figure 5B), confirming that this phenotype is associated with the *TOPO3α* deficiency. The results were obtained from three complemented clones (−/−/+) and two cultures each of wild type and *topo3α* mutant.

*RAD51*-dependent recombination intermediates accumulate in *top3* mutants and the removal of persistent intermediates requires the cleavage activity of Top3 [57], [58]. We examined whether the hyper-switching phenotype of *topo3α^−/−^* is dependent on *RAD51*. Both *RAD51* alleles were sequentially deleted in the wild-type and *topo3α^−/−^* strains. We analyzed four independent cultures of *rad51^−/−^* and two of *topo3α^−/−^ rad51^−/−^*. *RAD51* deletion reduced the switching frequency of the wild type by 2-fold and abolished the hyper-switching phenotype of *topo3α^−/−^* (Figure 5C). Collectively, we concluded that TOPO3α functions as an important regulatory factor for recombination-mediated *VSG* switching and that, in the absence of TOPO3α, recombinogenic structures may accumulate between the active ES and *VSG* donors, and could then be resolved to give rise to switched variants.

### *T. brucei* TOPO3α suppresses *VSG* GC and crossover

**Figure 6:**
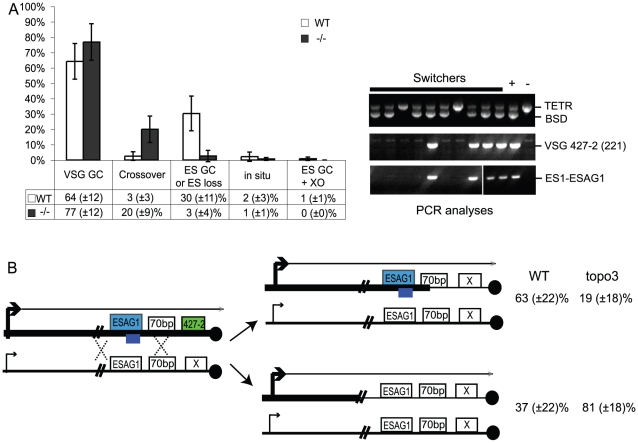
Analyses of switched variants. (A) TOPO3α suppresses *VSG* GC and crossover. 296 cloned switchers from three independent cultures of wild type and *topo3α^−/−^* were examined. The percentage of each mechanism (*VSG* GC, crossover, ‘ES GC or ES loss’, *in situ*, and ES GC+crossover) was plotted. White bars are wild type and dark grey bars are *topo3α* mutants. PCR results from several switchers are shown in right. *TETR* region was used as an internal PCR control, as all strains used in this study contain *TETR*. *VSG*-GC switchers should be *BSD^+^*, *VSG* 427-2^−^, and either *ESAG1^+ or −^*. ‘ES GC or ES loss’ switchers should be *BSD^−^*, *VSG* 427-2^−^, and *ESAG1^−^*. Crossover should be *BSD^+^*, *VSG* 427-2^+^, and *ESAG1^+^*. *In situ* should be *BSD^+^*, *VSG* 427-2^+^, and *ESAG1^+^*. The results are also summarized in Table 2. (B) TOPO3α specifically regulates the ES-associated *VSG* switching. Diagram shows relations between the presence of ES1-specific *ESAG1* and the location of recombination that occurred or resolved in *VSG*-GC switchers. Blue lines under *ESAG1* box indicate a region amplified by PCR. Black circles are telomere repeats.

In other organisms, Top3 defects are associated with elevated crossover as well as hyper-recombination [32]–[34], [45], [46]. To learn how individual switchers had undergone antigenic variation, we analyzed total 296 cloned switchers. The rationales for the double marking of parental cells are as follows (Figure 4). First, switchers can be effectively counter-selected using GCV (Figure 4 and 5). Second, transcription is initiated at silent ESs but elongation is prematurely terminated [59]: genes that are located closer to silent ES promoters are not completely silenced. Therefore, *in-situ* switchers can be distinguished from recombination-mediated switchers using different concentrations of blasticidin. Based on our titration for blasticidin concentration, *in-situ* switchers can grow in 5µg/ml blasticidin but not in 100 µg/ml, while ES gene conversion (ES GC) switchers cannot grow in either concentration. *VSG* gene conversion (*VSG* GC) and *VSG*-exchange (crossover) switchers will be resistant to 100 µg/ml blasticidin, and these alternatives can be distinguished by the absence or presence of *VSG* 427-2, respectively, which can be analyzed by PCR. The strategies to score individual switching mechanisms are summarized in Table 1 and examples of PCR analyses are shown in Figure 6A (right).

**Table 1:** Strategies to score switching mechanisms by blasticidin sensitivity and PCR.

BSD 5µg/ml	BSD 100µg/ml	*BSD* PCR	*VSG* 427-2 PCR	ES1-*ESAG1* PCR	*VSG* 427-2 Downstream PCR	Switching mechanism
+	+	+	−	−		*VSG* GC, upstream of *ESAG1*
				+	−	*VSG* GC at 70-bp repeat by BIR
					+	*VSG* GC at 70-bp repeat by GC
+	+	+	+			Crossover
+	−	+	+			*In situ*
−	−	−	−			ES GC or ES loss*

*ES loss associated with multiple events potentially including *in-situ* switching.

We analyzed cloned switchers isolated from six independent cultures and were able to discriminate among the alternative switching mechanisms. The results are summarized in Table 2 and Figure 6A. Switchers from cultures 1 and 2 were isolated by the column method and switchers from culture 3 by directly plating in GCV. Switching occurred largely by gene conversion (Figure 6A). In both wild type and *topo3α* mutants, 64∼77% of switching exploited *VSG* GC. Crossovers were rare in wild type (∼3%) but, on average, 20% of switchers exchanged their *VSG*s in *topo3α^−/−^*. These data suggest that, in the absence of TOPO3α, recombination intermediates may be accumulated and these could be repaired mostly by duplicative *VSG* GC and crossover.

**Table 2:** Summary of switching mechanisms in wild-type and *topo3α^−/−^* cells indicating the total number of switchers in each culture and the numbers of switchers assigned to different switching mechanisms.

Genotype (Culture #)	Total	*VSG* GC**	Crossover	ES GC or ES loss*	*In situ*	ES GC+Crossover
*WT* (#1)	83	67 (30)	1	13	0	2
*WT* (#2)	37	21 (12)	0	16	0	0
*WT* (#3)	47	26 (23)	3	15	3	0
*topo3α^−/−^* (#1)	25	23 (0)	2	0	0	0
*topo3α^−/−^* (#2)	53	40 (14)	13	0	0	0
*topo3α^−/−^* (#3)	51	32 (7)	14	4	1	0

*ES loss associated with multiple events potentially including *in-situ* switching.

**The numbers of *VSG*-GC switchers that recombined at the 70-bp repeats are indicated in parentheses.

In a previous study designed to examine *in-situ* switching, using a cell line with *TK* marker inserted next to the active ES promoter, frequent loss of entire active ES was observed. This could be caused by duplicative transposition of a silent ES (ES GC) or by deletion of the active ES coupled with transcriptional activation of a silent ES [60]. In our experiments, ES GC and ES loss cannot be distinguished, as switchers that lost both *BSD* and *VSG* 427-2 genes could arise either by duplicative transposition of a silent ES or by ES breakage coupled with an ES transcriptional switch. The ‘ES GC or ES loss’ events were rather frequently detected in wild-type cells (average ∼30%), while they were either not detected (culture 1 and 2) or detected at a low frequency (4 our of 51 cloned switchers in culture 3) in the absence of *TOPO3α* (Figure 6A and Table 2). Interestingly, *RAD51* deletion significantly decreased ‘ES GC or ES loss’ frequency (unpublished data), indicating that ‘ES GC or ES loss’ events are mainly under the control of *RAD51*-dependent recombination.

We noticed that some switched variants had growth disadvantages. Depending on how long it took to saturate the medium, wild-type switchers were categorized as ‘fast’, ‘medium’ or ‘slow’. ‘ES GC or ES loss’ switchers were prevalent in clones that grew up more slowly (data not shown). The functions of *ESAG*s are mostly unknown, but expressing different ESAGs might be advantageous when entering different hosts [61]. The slower-growth phenotype of some of these switchers may reflect impaired function of one or more *ESAGs* in the bovine serum-containing culture medium, which appears to favor stable transcription of the *VSG* 427-2-containing ES1.

*In-situ* switchers were rare in our assay. This phenotype is different from previous reports [24], [62], for reasons we do not understand. In our hands, *in-situ* switchers generally grew slower than *VSG*-GC switchers, so *VSG*-GC switchers would quickly take over the switched population if it was initially mixed, although this is unlikely because our switching population was initiated at 500–1000 cells/ml, while it was at 5,000–10,000 cell/ml in previous assays. Before this seeding, cells were grown in the presence of drugs that prevented switching.

### *T. brucei* TOPO3α specifically regulates ES-associated *VSG* switching

The 70-bp repeat unit has been proposed to be a recombination hot spot, possibly as a potential target for a site-specific endonuclease playing a similar role to that of the HO-endonuclease in yeast. Such an endonuclease has not been identified in trypanosomes. The 70-bp repeats could serve as switching hot-spots because of their structural features [63], rather than require cleavage by a specific endonuclease. Early experiments suggested that the overall *VSG* switching-frequency was not reduced in the absence of 70-bp repeats or by inversion of the repeats although, when present in the correct orientation, the repeats were used more than 10% of the time [62]. More recently, however, it has been shown that the 70-bp repeats of the actively transcribed ES are prone to break, which could induce recombination-mediated switching, and that the switching frequency was greatly increased when breaks were experimentally induced at the 70-bp repeats, but not when induced elsewhere in the ES or in the absence of 70-bp repeats [56].

We mapped the region where the recombination occurred (or resolved) in the *VSG*-GC switchers from wild type and *topo3α* mutants, to learn whether the 70-bp repeat unit is the hot spot of duplicative *VSG* GC and whether TOPO3α can redirect this preference. *ESAG1* genes are located immediately upstream of the 70-bp repeats, and their sequence polymorphisms allowed us to design ES1-specific-*ESAG1* oligonucleotides for PCR analysis. PCR results from several *VSG*-GC switchers were shown in Figure 6A (right). The presence of ES1-specific *ESAG1* in *VSG*-GC switchers indicates that gene conversion occurred at 70-bp repeat regions, and its absence indicates that recombination occurred upstream of *ESAG1* (Figure 6B). Crossover and ‘ES GC or ES loss’ switchers were used to verify that the PCR primer set was amplifying only the ES1-specific *ESAG1* gene. The ES1-specific *ESAG1* was lost in all ‘ES GC or ES loss’ switchers but was detected in all crossover switchers examined, as expected. The ES1-specific *ESAG1* gene was amplified in ∼63% of *VSG*-GC switchers in wild-type cells but ∼81% of *VSG*-GC switchers lost the ES1-specific *ESAG1* gene in *topo3α^−/−^*, indicating that, in the absence of TOPO3α, the active ES recombined mostly with silent ESs upstream of *ESAG1*, rather than within the 70-bp repeats, but not with minichromosomal or telomere-distal *VSG*s. We concluded that the 70-bp repeat region is an important but not an essential element for recombination-mediated switching. Gene conversion upstream of 70-bp repeats, at *ESAG2*, has also been reported [64]. The primary function of TOPO3α may be to prevent accumulation of recombination intermediates constantly arising between the active and silent ESs, to maintain the integrity of ESs.

Recombination by a one-strand invasion event could replace *VSG*s by break-induced replication (BIR) [56]. Alternatively, a second strand invasion at homologous sequences within or downstream of the *VSG* could generate *VSG*-GC switchers. Duplication of a telomere-distal *VSG* into an active ES is a relatively rare event, at least in the modest extent to which switching events have been characterized experimentally, but it appears to serve as an important switching mechanism in later stage of infection and as a mechanism to further expand the expressed *VSG* repertoire [22], [65], [66]. The few telomere-distal *VSG* arrays so far characterized contain only short stretches of 70-bp repeats but lack telomeric repeats. To determine how *VSG* GC occurred, we analyzed the sequences downstream of the 3′ homology region of *VSG* 427-2 by PCR in all *VSG*-GC switchers (Supporting Figure S3). If the second strand invaded at this 3′ homology region, downstream sequences should be unchanged. We found, however, that the ES1-specific downstream sequences were lost in all the *VSG*-GC switchers obtained from wild-type and *topo3α* cells, indicating that *VSG*-GC switchers were most likely repaired by BIR, consistent with a recent report [56], and that internal-*VSG* duplication is extremely rare. PCR results from a selection of *VSG*-GC switchers were shown in Figure S3.

To confirm the duplicative translocation of newly expressed *VSG*s to the *VSG* 427-2 ES and to examine whether minichromosomal *VSG*s contribute to antigenic switching, 32 *VSG*-GC switchers from wild-type cells were further analyzed. Minichromosomes terminate with telomeres, *VSG*s and 70-bp repeats. Gene conversion with minichromosomal *VSG*s occurs frequently [56], but only when recombination is initiated at the 70-bp repeats. Therefore, we cloned and sequenced newly activated *VSG*s from *VSG*-GC switchers that utilized 70-bp repeats. From 32 switchers that had undergone at least one type of switching, *VSG* GC at the 70-bp repeats, we obtained eight different newly activated *VSG*s (Supporting Figure S5, left). It is possible that we have underestimated the number of independent switching events as these switchers may have used different sequences within or near the 70-bp repeats, which should be counted as independent. Some switchers might have arisen earlier than others, for examples *VSG* 427-32, as these were presented more often than others. Among these eight newly expressed *VSG*s, four were novel *VSG*s, 427-32, 33, 34 and 35, full or partial sequences of which can be found in the following website (http://tryps.rockefeller.edu). Switchers expressing *VSG*s 427-3, 11, 32, 33 and 35 were examined by rotating agarose gel electrophoresis (RAGE) and Southern blot [56]. As shown in Supporting Figure S5 (right panel), *VSG* 427-2 was lost in all the switchers and all newly expressed *VSG*s were duplicated and translocated to the 427-2 ES, except for 427-33, an intermediate chromosomal (IC) *VSG*. The original copy of 427-33 may be lost after recombination. *VSG*s 427-32 and 35 came from megabase chromosomes (MBC). We have not isolated any minichromosomal *VSG*s in these switchers, indicating that recombination between ES-associated *VSG*s was the major source for *VSG* switching.

## Discussion

Repair by recombination serves to preserve genome integrity and can either homogenize or diversify genetic information, occasionally causing detrimental outcomes or benefiting certain organisms by providing adaptation systems to escape lethal situations. African trypanosomes escape the host immune response through a mechanism known as the antigenic variation. Here, we report that *T. brucei* TOPO3α, a member of a potential *T. brucei* RecQ-Top3-Rmi1 (RTR) complex, takes an important part in the regulation of recombination-mediated antigenic variation. Our results reveal a complex mechanism that has to balance ES integrity and *VSG* diversity to maximize the survival of a trypanosome population by suppressing crossovers on one hand and by promoting duplicative *VSG* gene conversions on the other.

### Mechanism of recombination-mediated antigenic switching and roles for TOPO3α

**Figure 7:**
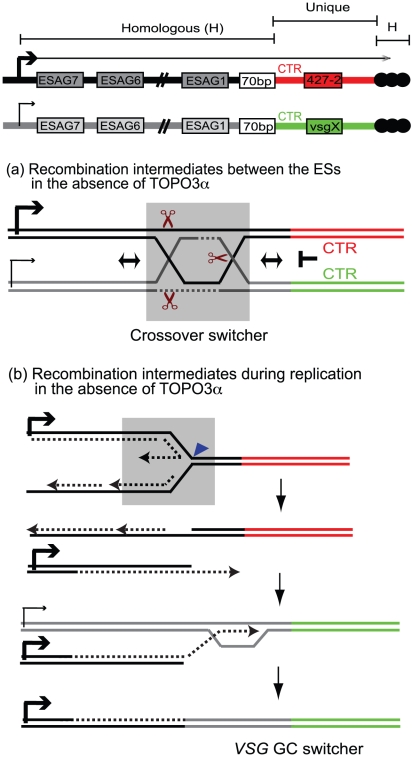
Mechanism of recombination-mediated antigenic switching and roles for TOPO3α. The diagram shows the active ES (ES1) expressing *VSG* 427-2 and a silent ES containing *vsg X*. *VSG* genes are located between 70-bp repeats and telomere repeats (black circles). The sequence of each *VSG* cassette, including CTR, *VSG*, and *VSG* downstream, is unique (red for *VSG* 427-2 and green for *vsg*X). The strong sequence similarities are present throughout these ESs. Holliday Junctions (HJs) or double HJs (dHJs) can form between these ESs but cannot migrate downstream of the 70-bp repeats. These intermediates have to be resolved before CTR or have to use telomere repeats or sequence homology within *VSG* cassettes to generate switched variants. The grey boxes (in (a) and (b)) include potential players that could generate switched variants in the absence of TOPO3α. (a) The dHJs can be efficiently processed by TOPO3α, generating non-crossover (no switching). However, in the absence of TOPO3α, HJ can be cleaved by resolvase (brown scissors) to generate non-crossover (no switching) and crossover products (crossover switchers). (b) Replication fork instability can accumulate recombination intermediates between sister-chromatids. In the absence of TOPO3α, these intermediates can be cleaved by MUS81, a 3′ flap endonuclease (blue triangle), and the broken leading strand can invade sister-chromatid to complete replication. Alternatively this can also invade a silent ES using their sequence homology and replicate to the end of the chromosome, generating *VSG* GC switchers.

As illustrated in Figure 7, ES structures seem to play a particular role in *VSG* switching. ES-associated *VSG* genes are located between the 70-bp and telomeric repeats. *ESAG*s and some pseudogenes are present upstream of the 70-bp repeats in all ESs, sometimes duplicated and sometimes missing [3], [9]. Strong sequence homologies are present throughout the ESs, with the exception of most of the *VSG* coding sequence and the immediately upstream ‘co-transposed region’ (CTR). *VSG* sequences are highly dissimilar except for ∼200-bp encoding the C-terminus and within the 3′ UTR [67]. The reason why every *VSG* cassette contains a unique CTR is unknown. The purpose of CTR could be to insulate the individuality of *VSG* cassettes, so that the *VSG* sequences can evolve separately from other regions in ESs, which maintain their sequences to serve for *VSG* recombination. When HR occurs, the CTR could block branch migration of HJ or dHJ downstream of the 70-bp repeats.

What roles does TOPO3α play in this scheme? Our study shows that TOPO3α deficiency increases *VSG* switching, especially *VSG* GC and crossover, and that the hyper-switching phenotype requires *RAD51*. The accumulation of toxic recombination intermediates accounts for the slow growth phenotype of yeast *top3* mutants, which is suppressed by mutations in *SGS1* or in the *RAD51*-pathway [43], [68], [69]. Recombination intermediates accumulate in cells over-expressing dominant-negative *Top3-Y356F* in response to methylmethane sulfonate in a *RAD51*-dependent manner [58]. The function of TOPO3α is not restricted to the 70-bp repeats in antigenic switching, as its absence appears to cause promiscuous recombination throughout the ESs. We therefore propose that TOPO3α removes recombinogenic structures constantly arising between ESs so as to maintain the albeit limited individuality of different ESs. In the absence of TOPO3α, recombination intermediates would accumulate during *VSG* switching and unresolved intermediates would have to be repaired either by GC associated with crossover or by placing a new duplicated *VSG* into the active ES by BIR (Figure 7).

Suppression of crossover in recombination-mediated *VSG* switching is an interesting result, considering that there are probably more than 200 potential *VSG* donors: ∼20 ESs with extensive sequence homology and ∼200 minichromosomal *VSG*s. Antigenic variation probably requires balancing preservation and variation of *VSG* information, but we cannot explain how suppression of crossover would be important for maintaining this balance. However, we think that by favoring duplicative GC over crossover, rather than crossover over GC, trypanosomes could slowly accumulate *VSG* diversity without abrupt loss of their functionalities, because duplicative GC requires *VSG* DNA synthesis, during which point mutations could be incorporated into newly synthesized *VSG*s, but *VSG* crossover does not require *VSG* DNA synthesis.

TOPO3α deficiency increased *VSG* GC far more than GC at the *URA3* locus (Figures 3 and 6). GC at these two loci is probably mediated by different pathways. Recombination at *URA3* locus would prefer flanking homologies, rather than BIR. In contrast, BIR would present a better option for *VSG* GC, as only one end homology appears to be involved (supporting Figure S3) [56]. It is possible that a second invasion could occur within the telomere repeats, but this is impossible to determine. The higher *VSG* GC rate could also be because the active ES is less stable than *URA3* locus. Alternatively, TOPO3α may specifically suppress BIR-mediated *VSG* switching. The role of TOPO3α in BIR has not been extensively characterized elsewhere. Our results show a novel function of TOPO3α in *VSG* switching, which could be an excellent system to study BIR.

### TbRTR complex and DSB-HR response in antigenic variation

DNA recombination involves many factors, of which only a few have been studied in the context of antigenic variation: RAD51, RAD51-related genes, BRCA2, KU70/80, MRE11, and MSH2/MLH1 [23]–[25], [29], [30], [70], [71]. Among these, only the deletion of *RAD51*, *RAD51-3*, and *BRCA2* decreased *VSG* switching, in wild-type cells that already had a very low switching rate.

Our findings on TOPO3α in *VSG* switching suggest potential roles for numerous DSB-HR response factors in antigenic variation. Two RecQ family helicases are annotated in the *T. brucei* gene database (http://www.genedb.org/genedb/tryp). Rmi1 is required to load Top3 onto the substrates and stimulate its activity through the physical interaction [72]. We have identified a *TbRMI1* homologue. All the phenotypes that we have examined in *Tbrmi1* mutants were identical to those in *topo3α* mutants (unpublished data). Therefore, we believe that RecQ, TOPO3α and RMI1 are likely to function as a complex in antigenic variation in *T. brucei*.

Synthetic-lethality screens with *sgs1* in budding yeast identified three pathways working in parallel with Sgs1 [73]; Mus81-Mms4, Slx1-Slx4, and Slx5-Slx8. Synthetic lethality of *sgs1 mus81* or *sgs1 mms4* requires HR factors [74]. Mus81-Mms4 is a structure-specific endonuclease that cleaves 3′ flap, replication fork, or HJ substrates [74]–[76]. Resolvase, an endonuclease, symmetrically cleaves HJs and the products can be resolved with crossover or non-crossover. Human and yeast resolvases have recently been characterized [77]. *MUS81* appears to be present in *T. brucei* but a resolvase remains to be identified. Although we do not yet have functional data for these proteins, we propose, based on the studies from other organisms, that the regulation of antigenic variation is similar to that of mitotic HR. When present, TOPO3α could dissolve dHJs to prevent the ES instability, consequently generating non-crossover recombinants (no switching). In the absence of TOPO3α, resolvase (Figure 7a, grey box) or MUS81 may cleave the accumulated recombination intermediates arising between the ESs and generate crossover switchers. Alternatively, stalled replication forks can be cleaved by MUS81 and the broken leading strand can invade a silent ES to generate *VSG*-GC switchers (Figure 7b, grey box).

Although *VSG* switching has similarities with mitotic HR, it appears that specific elements are present for its regulation. A hyper-recombination phenotype does not always correlate with hyper-switching phenotype. The mismatch repair (MMR) pathway can abort recombination during strand exchange between non-identical substrates and *mmr* mutants can increase recombination frequency (reviewed in [78]). Consistent with their roles in repair and recombination, *Tbmsh2* or *Tbmlh1* mutants increased recombination frequency but did not change switching frequency [71]. Recombination is closely linked with DNA replication and checkpoint pathways as well [32], [57], [58], [79]. Therefore, we believe that roles for DNA replication, checkpoint, and recombination factors and their interactions need to be determined to fully understand the mechanisms of antigenic variation.

Measuring *VSG* switching has, until now, been time-consuming and not very reproducible. Our new switching assay circumvents previous technical difficulties and can effectively assign specific roles to individual proteins.

### What triggers antigenic switching

It has recently been shown that a DSB introduced at the active 70-bp repeats by the I-*Sce*I endonuclease causes a 250-fold increase in *VSG* switching and that the DSBs were repaired by BIR [56]. However, it is unknown whether the *VSG* switching is activated by targeted DSBs or by random chromosomal breaks, or whether recombinogenic ssDNA is a primary cause for the initiation of *VSG* switching. HR can be instigated by many different sources; random breaks, endonuclease cleavage at specific target sites, replication fork instability, unusual secondary DNA structure, or transcription.

The Mre11 complex, which consists of Mre11, Rad50, and Xrs2 (NBS1 in mammals), plays a central role in the DSB-HR response [26]–[28]. MRE11 deficiency, however, did not change the *VSG* switching frequency [29], [30], promoting the idea that ssDNA regions may generate recombinogenic structures for the initiation of switching. Uncoupling of leading and lagging strand DNA synthesis caused by DNA lesions can destabilize a replication fork, leaving ssDNA gaps behind the fork, which could be processed into recombinogenic structures. If an ssDNA gap is a major trigger for recombination-mediated switching, switching frequency should increase in cells suffering from replication challenge. To address this issue, we treated cells with aphidicolin, an inhibitor of lagging strand DNA synthesis, and HU, and measured the switching frequency in parallel (Supporting Figure S4). Cells were treated with the drugs at a sub-lethal dose to exclude a possibility of chromosome break-induced switching. No significant correlation was observed between these treatments and switching frequency. Therefore, an ssDNA gap may not be a major initiating factor for *VSG* switching. Rather, random breaks might be responsible for switching induction, consistent to a previous study [56]. However, it is still difficult to rule out the possibility that an ssDNA gap triggers switching, as ssDNA gaps might not be extensive enough to create recombinogenic structures at the low doses of aphidicolin or HU. The best way to test this hypothesis would be to use conditional mutants associated with replication defects. Unfortunately, we do not yet have such genetic tools, as nuclear DNA replication has not been studied in *T. brucei*.

A high transcription level can stimulate recombination, a mechanism known as transcription-associated recombination (TAR) (reviewed in [80]). Transcription has been shown to promote recombination in *T. brucei* [81], [82]. Interestingly, it was shown in budding yeast that transcription- and DSB-induced recombination events were similar, indicating that transcription affects only the initiation of recombination, not the mechanism of recombination [83]. ssDNA regions exposed in the active ES during transcription could be readily accessible by recombination factors. Alternatively, transcription-replication collision causes replication fork stalling, which could also induce switching. Studies of mammalian cells have shown that TAR is dependent on replication [84], and that transcription increases recombination frequency when a replication fork converges with transcription [85]. The active ES is more fragile than silent ESs [56]. The high level of transcription may explain why the active ES breaks more frequently, and this may induce *VSG* switching.

The 70-bp repeat has been proposed to be a potential endonuclease target site to induce switching, but such an enzyme has not been found. Instability of the 70-bp repeat [63] may play a role in the initiation of switching and could lead to template switching. However, according to our results and previous studies [62], [64], switching is not completely dependent on the 70-bp repeats. With the available data, it would be reasonable to conclude that random breaks may occur throughout the active ES but more frequently at 70-bp repeats, and these could initiate various switching events.

Gene conversion is used by several other pathogens, including *Borrelia hermsii* and *Anaplasma marginale*, as an evasion mechanism [10], [86]. Our study suggests that exploring how trypanosomes manipulate the HR machinery to gain advantage against their host's immunity, while successfully preserving their genomes, may reveal weaknesses that can be exploited to control infectivity and virulence.

## Materials and Methods

### *Trypanosome* strains and plasmids

*Trypanosoma brucei* bloodstream forms (strain Lister 427 antigenic type MITat1.2 clone 221a (VSG 427-2)) were cultured in HMI-9 at 37°C. The cell lines constructed for this study are listed in Supporting Table S1, and they are of ‘single marker’ (SM) background that expresses T7 RNA polymerase and Tet repressor (*TETR*) [87]. Stable clones were obtained and maintained in HMI-9 media containing necessary antibiotics at the following concentrations, unless otherwise stated: 2.5µg/ml, G418 (Sigma); 5µg/ml, blasticidin (Invivogen); 5µg/ml, hygromycin (Sigma); 0.1µg/ml, puromycin (Sigma); 1µg/ml, phleomycin (Invivogen). Plasmids used for this study are listed in Supporting Table S2.

### Construction of *topo3α^−/−^* cell line and removal of markers using Cre recombinase-loxPs (Supporting Figure S2)

*TOPO3α* genes were sequentially deleted using deletion-cassettes containing either puromycin or hygromycin-resistance gene fused with *HSVTK*, *Herpes simplex* virus thymidine kinase (*TK*), *PUR-TK* and *HYG-TK*. These fusion genes are flanked by loxP sites so that the markers can be removed by transient expression of Cre recombinase (pLew100-Cre). The entire open reading frame (ORF) of *TOPO3α* was deleted by transfecting ‘single marker’ (SM) cells with a deletion-cassette that was amplified with primer 35 and 36 using pHJ18 (*PUR-TK*) as a template. Primer 35 and 36 contains 70 nt homologies to the target sites. This *topo3α* ‘single knock-out’ cells (*sKO*, HSTB-97) were used to PCR amplify a cassette containing a marker (*PUR-TK*) along with 453 nt upstream and 402 nt downstream sequences of *TOPO3α* gene. The PCR fragment was inserted into pGEM-easy-T vector by TA cloning to create pHJ63. pHJ64 was constructed by replacing a *PUR-TK* marker with a *HYG-TK* from pHJ17. *topo3α* ‘double knock-out’ (dKO) was generated by transfecting *Not*I-digested pHJ64 into *topo3α sKO*, HSTB-97. Deletion of both *TOPO3α* alleles was confirmed by PCR analyses.

To remove the selection markers, *topo3α dKO* cells were transfected with pLew100-Cre to transiently express Cre-recombinase, and the cells that lost both *HYG-TK* and *PUR-TK* were selected in 50µg/ml ganciclovir (GCV). Loss of markers was confirmed by resistance to puromycin and hygromycin, and by PCR analysis. The sequences of primers used here are available upon request.

### Recombination assay

pLHTL-pyrFE [48]-linearized by *Pvu*II digestion was transfected into wild-type (HSTB-188) and *topo3α^−/−^* (HSTB-328 and HSTB-330) cells, to replace one allele of *TbURA3* with *HYG-TK*. The integration was confirmed by PCR analysis with primers 48 and 49. Three or five independent *HYG^R^* clones from wild-type or *topo3^−/−^* cells were analyzed. Cells were grown in the absence of hygromycin for 2 days to allow recombination to occur. Approximately 500,000 cells were diluted in HMI-9 media containing 30 µg/ml GCV or 6 µg/ml FOA, and distributed into 96-well plates. Yellow wells (phenol red indicating acidification due to growth) containing *GCV^R^* or *FOA^R^* cells were counted after 7–8 days of incubation and the GC frequency was determined. The sequences of primers used for genotyping are available upon request.

### Switching assay and analyses of switchers

To create a doubly-marked switching reporter strain (Figure 4), pHJ23 was linearized by *Kpn*I-*Not*I digestion and integrated downstream of the ES1 promoter, to confer resistance to blasticidin. These cells were then marked with *PUR-TK* at the 3′ end of 70-bp repeats by transfecting a PCR-amplified *PUR-TK* cassette. Ten µg/ml of puromycin, 100 times higher than normal usage, was added to select clones targeted specifically at the active ES. When determining switching frequency, the parental cells were maintained in the presence of blasticidin and puromycin to exclude switchers from the starting population. Cells were then allowed to switch in the absence of selection for 3–4 days. Switchers were enriched using a MACS [56]. Flow-through enriched with switchers was collected and serially diluted in media containing 4 µg/ml GCV, and distributed into 96-well plates. The switching frequency was determined by counting *GCV^R^* clones. Alternatively, switching frequency was determined without the column enrichment step. Cells were diluted in GCV-containing media and directly distributed into 96-well plates. Non-switchers that carry spontaneous mutation(s) in *TK* gene but not in *PUR* were ruled out by examining puromycin resistance. Non-switchers that carry mutations in *PUR* and *TK* were ruled out by western blot analysis using antibodies against VSG 427-2.

To determine switching mechanisms, cloned switchers were analyzed for blasticidin sensitivity at 5 µg/ml and 100 µg/ml concentrations. Genomic DNA was prepared from 296 switchers and PCR-analyses were performed at four regions: *BSD*, *VSG* 427-2, *ESAG1*, and *VSG* 427-2 downstream. The primer set designed for *BSD*-PCR can also amplify *TETR* (Tet Repressor) gene, which was used as a control for PCR analyses. The sequences of primers used here are available upon request.

### Analysis of sensitivity to genotoxic agents

Wild type (SM), *topo3α^−/+^* (*HSTB-97*), and *topo3α^−/−^* (*HSTB-226* and *HSTB-227*) cells were incubated with indicated concentration of phleomycin for 24 hours. The same number of cells was distributed into 96-well plates. All the plating was duplicated. The wells that contain viable cells were counted after 7–8 days of incubation at 37°C and the viability was calculated by normalizing to untreated samples. Sensitivity to HU and aphidicolin was determined similarly. Cells were incubated with HU or aphidicolin for 2 or 3 days. The viability was calculated by normalizing to untreated samples.

### Gene accession numbers

Database ID numbers (http://www.genedb.org and http://tritrypdb.org) for *TOPO3α* discussed in this paper are Tb11.01.1280, LmjF36.3200 and Tc00.1047053511589.120. What we refer to as *TbURA3* is the bifunctional orotidine-5-phosphate decarboxylase/orotate phosphoribosyltransferase Tb927.5.3810.

## Supporting Information

Table S1 Strains used in this study
(0.05 MB DOC)

Table S2 Plasmids used in this study
(0.05 MB DOC)

Figure S1 Alignment of *T. brucei*, *T. cruzi* and *L. major* TOPO3α. The colored boxes indicate domains found in SMART (Simple Modular Architecture Research Tool) domain search: yellow box, TOPRIM (topoisomerase-primase) domain; purple, TOP1Bc (bacterial DNA topoisomerase I ATP-binding domain); red, TOP1Ac (bacterial DNA topoisomerase I DNA binding domain); green, Zf-C4 (zinc-finger domain). The catalytic tyrosine (Y) is written in yellow. Four cysteine residues are written in red in green box. Gene numbers are Tb11.01.1280, LmjF36.3200 and Tc00.1047053511589.120.
(0.52 MB TIF)

Figure S2 Construction of *topo3α* ^−/−^ cell line and removal of markers using Cre recombinase-loxPs. *TOPO3α* genes were sequentially deleted using deletion-cassettes containing *PUR-TK* and *HYG-TK*. These fusion genes are flanked by loxP sites so that the markers can be removed by transient expression of Cre recombinase (pLew100-Cre) [48].
(0.36 MB EPS)

Figure S3 Switching occurred by BIR in *VSG*-GC switchers. Diagram shows two scenarios of how new *VSG* can be duplicated and placed in the active ES. The first strand invasion should occur upstream of CTR and this can replicate all the way to the end of the chromosome or recombine a second time at the homology regions present at the C-terminus or in the 3′ UTR of *VSG* 427-2 (221) (3′ homology region). To distinguish these possibilities, the downstream region specific for *VSG* 427-2 (221) was analyzed in all the switchers by PCR. 12 switchers (2 crossover and 10 *VSG*-GC switchers) are shown as representatives. Black circles are telomere repeats. Blue lines next to telomere repeats indicate a region analyzed by PCR.
(0.36 MB EPS)

Figure S4 Switching frequency was not affected by aphidicolin or HU treatments. (A) Wild-type cells were treated with the indicated concentrations of aphidicolin and percent viability was determined by normalizing to the untreated sample. (B) Switching frequency of aphidicolin-treated cells was measured in parallel, by directly plating in GCV-containing media without enrichment. (C) Wild-type cells were treated with 0.01 mM HU, and *topo3α* cells were treated with 0.01 mM HU or 1 ng/ml aphidicolin (APH) for 3 days. Percent viability was determined by normalizing to untreated sample. (D) Switching frequency of HU or aphidicolin-treated wild-type or mutant cells was measured in parallel by the column-enrichment method.
(0.34 MB EPS)

Figure S5 Cloning newly activated *VSG*s. Total mRNA was extracted from *VSG*-GC switchers that utilized 70-bp repeats for *VSG* recombination. cDNA was amplified using a reverse-transcriptase and oligo dT_20_ (Stratagene). Newly expressed *VSG*s were amplified using specific oligos that anneal to the spliced leader and to 16-mer sequences present in all *VSG* transcripts, and sequenced. Eleven switchers expressing 427-3 (224), 427-11 (bR2), 427-32, 427-33 or 427-35 were further analyzed to confirm duplicative translocation of new *VSG*s to the *VSG* 427-2 expression site by rotating agarose gel electrophoresis (RAGE) and Southern blotting [56], using probes specific to *VSG*s 427-2 (221), 427-3, 427-11, 427-32, 427-33, or 427-35. Abbreviations: MBC (megabase chromosome), IC (intermediate chromosome), MC (minichromosome), and P (parental strain expressing VSG 427-2). Arrowheads indicate translocation of newly activated *VSG*s to ES1.
(0.89 MB TIF)
