# Supplementary material for: TOPO3α Influences Antigenic Variation by Monitoring Expression-Site-Associated VSG Switching in Trypanosoma brucei
Source: PLoS Pathog. 2010 Jul 8;6(7):e1000992. doi: 10.1371/journal.ppat.1000992 (PMC2900300; doi:10.1371/journal.ppat.1000992)
Supplement: Table S1 — Strains used in this study (0.05 MB DOC) [file ppat.1000992.s001.doc]

Table S1. Strains used in this study

| Names | Genotypes | Manipulations |
| --- | --- | --- |
| *SM* | *WT* | *T7 RNA polymerase* and *Tet repressor (TETR):: NEO* [87] |
| *HSTB-97* | *topo3α^-/+^* | *topo3α∆loxP-PUR-TK-loxP/+* |
| *HSTB-226* | *topo3α^-/-^* | *topo3α∆loxP-PUR-TK-loxP/ topo3α∆loxP-HYG-TK-loxP* |
| *HSTB-227* | *topo3α^-/-^* | *topo3α∆loxP-PUR-TK-loxP/ topo3α∆loxP-HYG-TK-loxP* |
| *HSTB-246* | *topo3α^-/-^* | *ES1 promoter::BSD, topo3α∆loxP-PUR-TK-loxP/ topo3α∆loxP-HYG-TK-loxP* |
| *HSTB-188* | *WT* | *ES1 promoter::BSD* |
| *HSTB-261* | *WT* | *ES1 promoter::BSD, 70-bp repeats-PUR-TK* |
| *HSTB-328* | *topo3α^-/-^* | *ES1 promoter::BSD, topo3α∆loxP/ topo3α∆loxP* |
| *HSTB-330* | *topo3α^-/-^* | *ES1 promoter::BSD, topo3α∆loxP/ topo3α∆loxP* |
| *HSTB-278* | *topo3α^-/-^* | *ES1 promoter::BSD, 70-bp repeats-PUR-TK, topo3α∆loxP/ topo3α∆loxP* |
| *HSTB-344* | *topo3α^-/-^* | *ES1 promoter::BSD, 70-bp repeats-PUR-TK, topo3α∆loxP/ topo3α∆loxP* |
| *HSTB-299* | *topo3α^-/-^ + TOPO3α* | *ES1 promoter::BSD, 70-bp repeats-PUR-TK, topo3α∆loxP/ topo3α∆loxP + TOPO3α-wt* |
| *HSTB-301* | *topo3α^-/-^ + TOPO3α* | *ES1 promoter::BSD, 70-bp repeats-PUR-TK, topo3α∆loxP/ topo3α∆loxP + TOPO3α-wt* |
| *HSTB-302* | *topo3α^-/-^ + TOPO3α* | *ES1 promoter::BSD, 70-bp repeats-PUR-TK, topo3α∆loxP/ topo3α∆loxP + TOPO3α-wt* |
| *HSTB-365* | *rad51^-/-^* | *ES1 promoter::BSD, 70-bp repeats-PUR-TK, rad51∆HYG/ rad51∆PHELO* |
| *HSTB-378* | *topo3α^-/-^ rad51^-/-^* | *ES1 promoter::BSD, 70-bp repeats-PUR-TK, topo3α∆loxP/ topo3α∆loxP, rad51∆HYG/ rad51∆PHELO* |
| *HSTB-383* | *topo3α^-/-^ rad51^-/-^* | *ES1 promoter::BSD, 70-bp repeats-PUR-TK, topo3α∆loxP/ topo3α∆loxP, rad51∆HYG/ rad51∆PHELO* |
