# Supplementary material for: TOPO3α Influences Antigenic Variation by Monitoring Expression-Site-Associated VSG Switching in Trypanosoma brucei
Source: PLoS Pathog. 2010 Jul 8;6(7):e1000992. doi: 10.1371/journal.ppat.1000992 (PMC2900300; doi:10.1371/journal.ppat.1000992)
Supplement: Table S2 — Plasmids used in this study (0.05 MB DOC) [file ppat.1000992.s002.doc]

Table S2. Plasmids used in this study

| Names | Inserts, targeting loci, and markers | Sources |
| --- | --- | --- |
| pHD309 | Vector with targeting sequence for *TUB* array with *HYG* marker |  |
| pLHTL-pyrFE | *HYG-TK* targeting at *TbURA3* | [48] |
| pLEW100-Cre | Cre-recombinase in expression vector | [48] |
| pHJ17 | *loxP-HYG-TK-loxP* | This study |
| pHJ18 | *loxP-PUR-TK-loxP* | This study |
| pHJ23 | *BSD* targeting downstream of ES promoter | This study |
| pHJ63 | *TOPO3α* deletion construct with  *loxP-PUR-TK-loxP* marker | This study |
| pHJ64 | *TOPO3α* deletion construct with  *loxP-HYG-TK-loxP* marker | This study |
| pHJ80 | *TOPO3α* integration at *TUB* array with *HYG* marker | This study |
| pSY1 | *RAD51* deletion construct with  *HYG* marker | This study |
| pSY23 | *RAD51* deletion construct with  *PHLEO* marker | This study |
