# Supplementary figures and images for: TOPO3α Influences Antigenic Variation by Monitoring Expression-Site-Associated VSG Switching in Trypanosoma brucei
Source: PLoS Pathog. 2010 Jul 8;6(7):e1000992. doi: 10.1371/journal.ppat.1000992 (PMC2900300; doi:10.1371/journal.ppat.1000992)

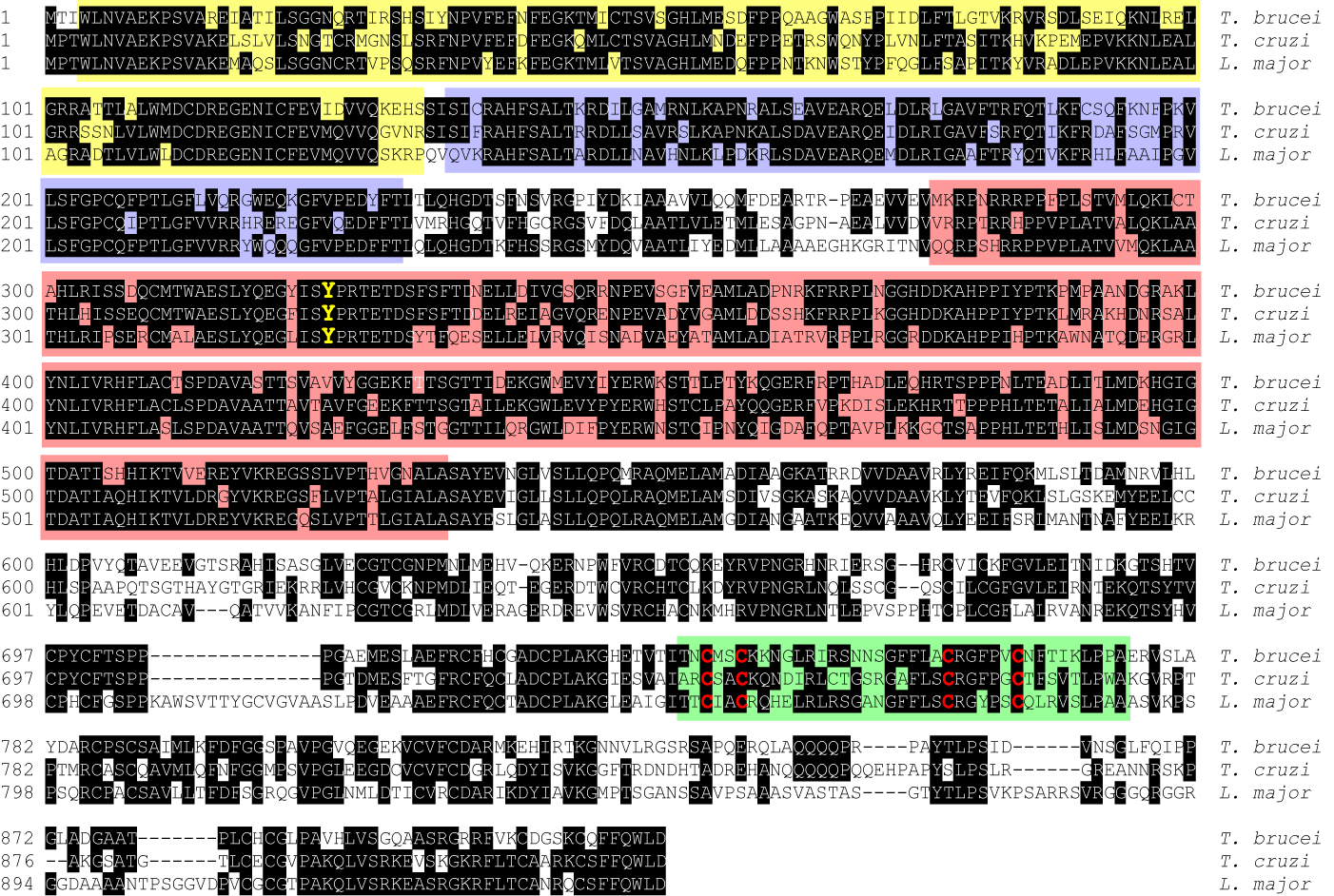

Supplement: Figure S1 — Alignment of T. brucei, T. cruzi and L. major TOPO3α. The colored boxes indicate domains found in SMART (Simple Modular Architecture Research Tool) domain search: yellow box, TOPRIM (topoisomerase-primase) domain; purple, TOP1Bc (bacterial DNA topoisomerase I ATP-binding domain); red, TOP1Ac (bacterial DNA topoisomerase I DNA binding domain); green, Zf-C4 (zinc-finger domain). The catalytic tyrosine (Y) is written in yellow. Four cysteine residues are written in red in green box. Gene numbers are Tb11.01.1280, LmjF36.3200 and Tc00.1047053511589.120. (0.52 MB TIF) [file ppat.1000992.s003.tif]

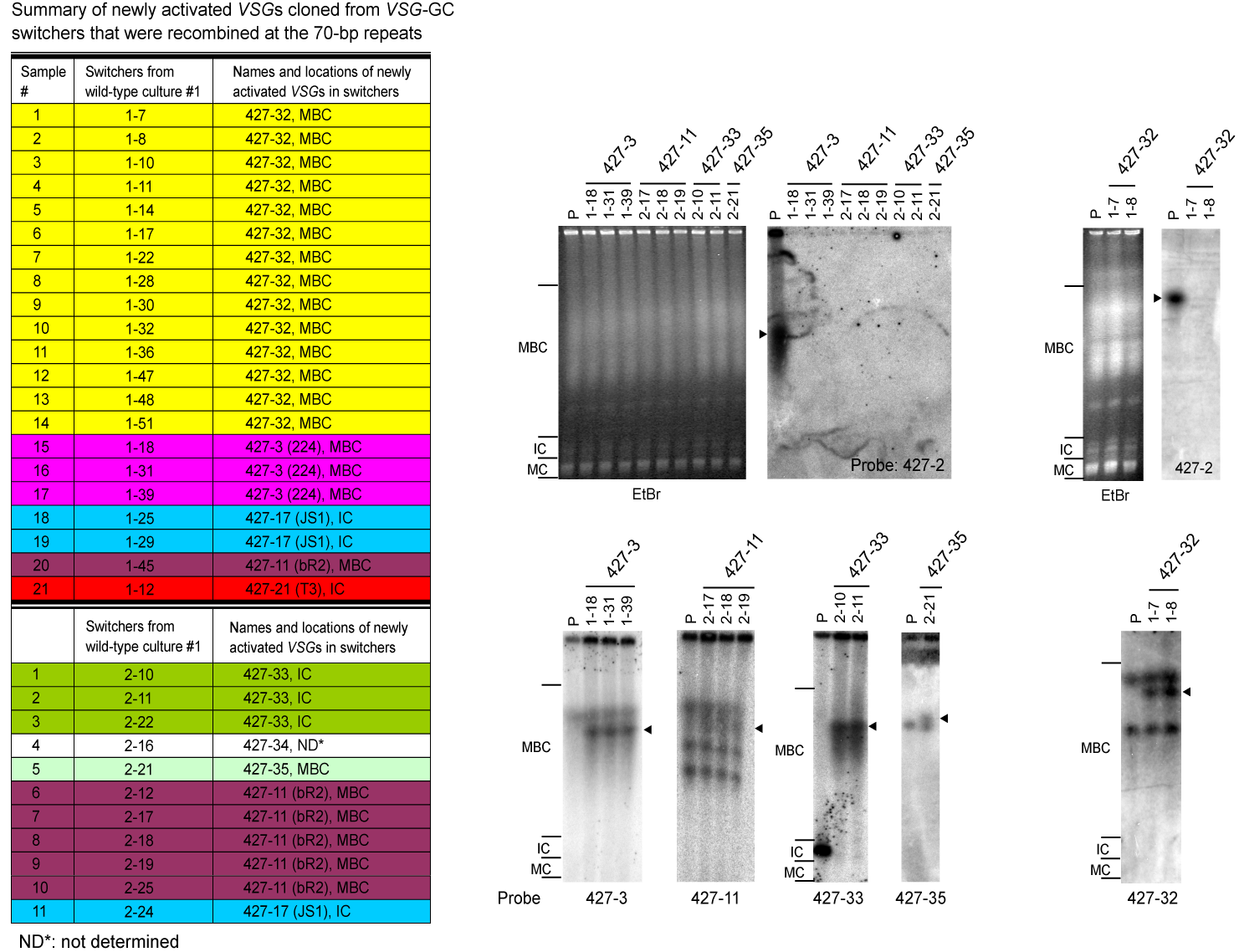

Supplement: Figure S5 — Cloning newly activated VSGs. Total mRNA was extracted from VSG-GC switchers that utilized 70-bp repeats for VSG recombination. cDNA was amplified using a reverse-transcriptase and oligo dT20 (Stratagene). Newly expressed VSGs were amplified using specific oligos that anneal to the spliced leader and to 16-mer sequences present in all VSG transcripts, and sequenced. Eleven switchers expressing 427-3 (224), 427-11 (bR2), 427-32, 427-33 or 427-35 were further analyzed to confirm duplicative translocation of new VSGs to the VSG 427-2 expression site by rotating agarose gel electrophoresis (RAGE) and Southern blotting [56], using probes specific to VSGs 427-2 (221), 427-3, 427-11, 427-32, 427-33, or 427-35. Abbreviations: MBC (megabase chromosome), IC (intermediate chromosome), MC (minichromosome), and P (parental strain expressing VSG 427-2). Arrowheads indicate translocation of newly activated VSGs to ES1. (0.89 MB TIF) [file ppat.1000992.s007.tif]
